# A Spontaneous Mutation in *Taar1* Impacts Methamphetamine-Related Traits Exclusively in DBA/2 Mice from a Single Vendor

**DOI:** 10.3389/fphar.2017.00993

**Published:** 2018-01-22

**Authors:** Cheryl Reed, Harue Baba, Zhen Zhu, Jason Erk, John R. Mootz, Nicholas M. Varra, Robert W. Williams, Tamara J. Phillips

**Affiliations:** ^1^Department of Behavioral Neuroscience and Methamphetamine Abuse Research Center, Oregon Health & Science University, Portland, OR, United States; ^2^Department of Genetics, Genomics and Informatics, University of Tennessee Health Sciences Center, Memphis, TN, United States; ^3^VA Portland Health Care System, Portland, OR, United States

**Keywords:** addiction, BXD RI strains, conditioned taste aversion, drinking, hypothermia, selected lines, substance use disorder, trace amine-associated receptor 1

## Abstract

Major gene effects on traits associated with substance use disorders are rare. Previous findings in methamphetamine drinking (MADR) lines of mice, bred for high or low voluntary MA intake, and in null mutants demonstrate a major impact of the trace amine-associated receptor 1 (*Taar1*) gene on a triad of MA-related traits: MA consumption, MA-induced conditioned taste aversion and MA-induced hypothermia. While inbred strains are fundamentally genetically stable, rare spontaneous mutations can become fixed and result in new or aberrant phenotypes. A single nucleotide polymorphism in *Taar1* that encodes a missense proline to threonine mutation in the second transmembrane domain (*Taar1*^*m1J*^) has been identified in the DBA/2J strain. MA is an agonist at this receptor, but the receptor produced by *Taar1*^*m1J*^ does not respond to MA or endogenous ligands. In the present study, we used progeny of the C57BL/6J × DBA/2J F2 cross, the MADR lines, C57BL/6J × DBA/2J recombinant inbred strains, and DBA/2 mice sourced from four vendors to further examine *Taar1*-MA phenotype relations and to define the chronology of the fixation of the *Taar1*^*m1J*^ mutation. Mice homozygous for *Taar1*^*m1J*^ were found at high frequency early in selection for high MA intake in multiple replicates of the high MADR line, whereas *Taar1*^*m1J*^ homozygotes were absent in the low MADR line. The homozygous *Taar1*^*m1J*^ genotype is causally linked to increased MA intake, reduced MA-induced conditioned taste aversion, and reduced MA-induced hypothermia across models. Genotype-phenotype correlations range from 0.68 to 0.96. This *Taar1* polymorphism exists in DBA/2J mice sourced directly from The Jackson Laboratory, but not DBA/2 mice sourced from Charles River (DBA/2NCrl), Envigo (formerly Harlan Sprague Dawley; DBA/2NHsd) or Taconic (DBA/2NTac). By genotyping archived samples from The Jackson Laboratory, we have determined that this mutation arose in 2001–2003. Our data strengthen the conclusion that the mutant *Taar1*^*m1J*^ allele, which codes for a non-functional receptor protein, increases risk for multiple MA-related traits, including MA intake, in homozygous *Taar1*^*m1J*^ individuals.

## Introduction

Substance use disorders and related traits have been characterized as complex, based on their influence by a combination of multiple genetic and environmental factors. Quantitative trait locus (QTL) mapping and genome-wide association studies have identified locations of influential gene variants. Understandably, because of the polygenic nature of these traits, the amount of genetic variance accounted for by each independent QTL has often been relatively small (Buck et al., 1997; Vadasz et al., 2007; Hall et al., 2013; Yazdani et al., 2015). Furthermore, though some genes have repeatedly been identified (Hall et al., 2013), candidate gene polymorphism associations for various addiction-related traits have not always been reproducible (Hart et al., 2013; Forero et al., 2015). A welcome exception is a recently mapped reproducible QTL that accounts for more than 50% of the genetic variance in methamphetamine (MA) intake (Belknap et al., 2013) in multiple sets of mouse lines selectively bred for high vs. low two-bottle choice MA consumption, collectively referred to as the MA drinking (MADR) lines. Among the genes within the confidence interval of this QTL on mouse chromosome 10 is the trace amine-associated receptor 1 (*Taar1*) gene. The direct agonist activity of MA at the receptor (TAAR1) expressed by this gene (Bunzow et al., 2001) led us to examine sequence databases for potential *Taar1* polymorphisms between the C57BL/6J and DBA/2J progenitor strains of the MADR lines. We found that the DBA/2J strain possesses a single nucleotide polymorphism (SNP) in *Taar1* at position 229 that encodes a missense proline (CCC) to threonine (ACC) mutation in the second transmembrane domain of the TAAR1 protein, compared to the B6 strain and 27 other inbred strains, including four wild-derived strains (Shi et al., 2016). This mutant allele has been named *Taar1*^*m1J*^. The impact of this amino acid change is to eliminate TAAR1 function (Harkness et al., 2015; Shi et al., 2016).

Although there could be more than one gene within the chromosome 10 QTL that influences level of MA intake, several lines of evidence support the hypothesis that this *Taar1* mutation is a quantitative trait nucleotide (QTN) with a major impact. First, selective breeding for MA intake is associated with a change in the frequency of the two *Taar1* allele types so that after five generations of selective breeding, the MA high drinking (MAHDR) mice are homozygously fixed for the *Taar1*^*m1J*^ allele, whereas the MA low drinking (MALDR) mice remain homozygous for the alternate, *Taar1*^+^, allele or are heterozygous (Harkness et al., 2015). MA intake is several-fold higher in MAHDR than MALDR lines (Wheeler et al., 2009; Shabani et al., 2011), and also in *Taar1* knockout mice, compared to their wildtype littermates (Harkness et al., 2015). Therefore, homozgosity for both the naturally occurring mutation and an engineered *Taar1* null mutation are associated with increased MA intake. Oral MA intake differences are not associated with differences in taste sensitivity (Wheeler et al., 2009; Shabani et al., 2011). In addition, MAHDR and *Taar1* knockout mice both exhibit reduced sensitivity to aversive and physiological effects of MA that could limit voluntary MA consumption (Wheeler et al., 2009; Shabani et al., 2011, 2012, 2016; Harkness et al., 2015), supporting a role for *Taar1* in these traits as well.

That the DBA/2J strain is the only one of the 27 strains we have examined that harbors the *Taar1*^*m1J*^ allele, raised the question of when this mutation arose. We were able to partially answer the question by sequencing recombinant inbred (RI) strains that were derived at different time periods from the F2 cross of the C57BL/6J and DBA/2J strains (collectively, the BXD RI strains). None of the strains created beginning in 1969, 1991, or 1998 possess the threonine substitution, whereas those produced beginning in 2008 do (Shi et al., 2016). These data confirm that the mutation is relatively recent in origin. However, we sought to narrow down when the mutation appeared during this 10-year span, because this information is important for the interpretation of data collected in DBA/2J mice before and after the mutation arose for which TAAR1 function is relevant. In addition, because DBA/2 mice can be purchased from various vendors, we sought to determine if the mutation is present in stocks at other vendors or only in those supplied by The Jackson Laboratory (Bar Harbor, ME and Sacramento, CA), the source of all genetic material so far examined for this mutation. In addition to answering these questions, we used several mouse models to further examine the association of *Taar1* genotype with the triad of genetically correlated MA-related traits: MA intake, MA-induced conditioned taste aversion (CTA) and MA-induced body temperature change (or thermal response). The models used were the C57BL/6J × DBA/2J F2 cross (F2 from this point forward), BXD RI strains, and DBA/2 stock from multiple vendors. Finally, we examined *Taar1* allele frequencies at earlier stages of selective breeding in the MADR lines to test the prediction that fixation of the *Taar1*^*m1J*^ allele occurs at an early stage of selection, consistent with a major single gene effect.

## Materials and methods

### Subjects

**Figure 1:**
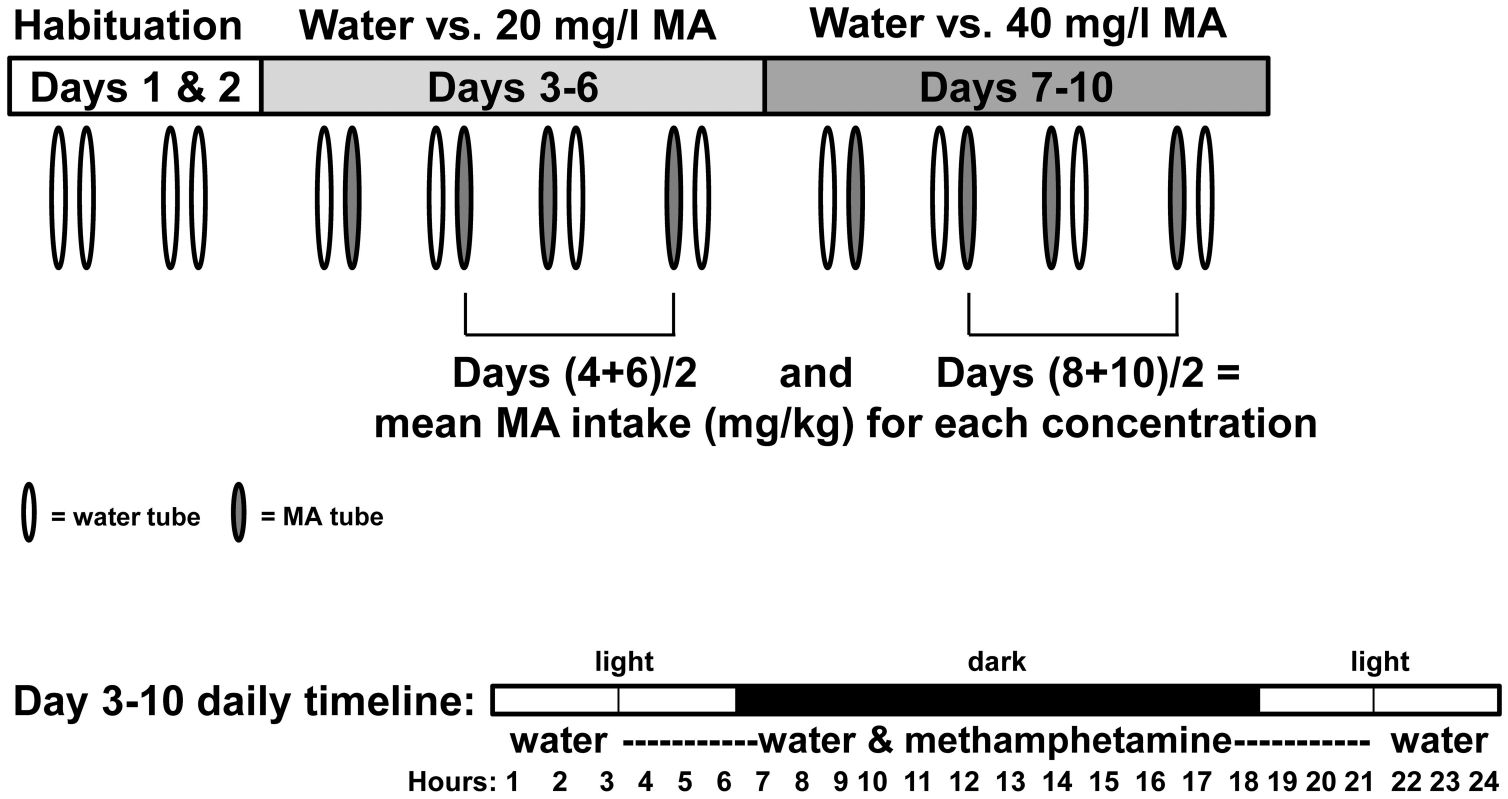
Diagram illustrating procedure for measuring two-bottle choice MA drinking. Mice were isolate-housed and given access to two, 25-ml graduated cylinders fitted with stoppers and sipper tubes for a 10-d period. On days 1 and 2, the tubes were filled with water. Mice were then given a choice between water and a solution of 20 mg MA/l tap water on days 3–6, and then 40 mg MA/l water on days 7–10. The MA tube was available for 18 h per day, beginning 3 h before the dark phase and ending 3 h into the light phase, as shown in the day 3–10 daily timeline. During the remaining 6 h, one tube containing water was available. The relative positions of the water and MA tubes on each day are indicated by tube coloration. Day 4 and 6 consumption values were averaged for each mouse and used to derive a mean MA intake value for the 20 mg/l concentration; Day 8 and 10 consumption values were averaged for each mouse and used to derive a mean MA intake value for the 40 mg/l concentration.

All experiments were performed in accordance with the National Institutes of Health Guidelines for the Care and Use of Laboratory Animals and were approved by the Institutional Animal Care and Use Committee of the VA Portland Health Care System (VAPORHCS). Mice were initially group-housed in polycarbonate shoebox cages (28.5 × 17.5 × 12 cm) with Bed-o'Cobs bedding (The Andersons, Inc., Maumee, OH) and maintained on a 12:12 h light:dark schedule, with lights on at 0600 h. Those that were not bred in-house were acclimated to the VAPORHCS for at least 2 weeks after shipping, prior to initiation of behavioral testing. Testing for MA-induced CTA and body temperature change was performed during the light phase, beginning no earlier than 2 h following lights on and no later than 2 h before lights off. The timing for assessment of MA intake is summarized in Figure 1. Mice had free access to laboratory rodent block food (Purina 5001or 5LOD PicoLab Rodent Diet; Animal Specialties, Woodburn, OR) and tap water at all times, except as described for specific procedures. See Table 1 for a summary of the genetic mouse models utilized.

**Table 1:** Mouse models utilized and their *Taar1* genotypes.

**Mouse model**	**Background genotype**	***Taar1* genotype**	**Phenotype tested**
C57BL/6J × DBA/2J F2	Mixed C57BL/6J and DBA/2J	*Taar1^**m*1*J*/*m*1*J**^, Taar1^**m*1*J*/+*^*, and *Taar1^+/+^*	MA intake
MAHDR selected line	Mixed C57BL/6J and DBA/2J	*Taar1^**m*1*J*/*m*1*J**^*	MA intake
MALDR selected line	Mixed C57BL/6J and DBA/2J	*Taar1^**m*1*J*/+*^* and *Taar1^+/+^*	MA intake
BXD RI strains	Mixed C57BL/6J and DBA/2J	*Taar1^**m*1*J*/*m*1*J**^* and *Taar1^+/+^*	MA intake, MA-induced CTA, thermal response to MA
DBA/2J inbred strain	Homozygous DBA/2J	*Taar1^**m*1*J*/*m*1*J**^*	MA intake, MA-induced CTA, thermal response to MA
DBA/2NCrl inbred strain	Homozygous DBA/2	*Taar1^+/+^*	MA intake, MA-induced CTA
DBA/2NHsd inbred strain	Homozygous DBA/2	*Taar1^+/+^*	MA intake, MA-induced CTA
DBA/2NTac inbred strain	Homozygous DBA/2	*Taar1^+/+^*	MA intake, MA-induced CTA, thermal response to MA

### F2 mice

For the creation of multiple serial replicate pairs of MADR selected lines that have been produced at 2-year intervals, the F2 cross of the C57BL/6J and DBA/2J strains were produced in the VAPORHCS animal facility and tested for two-bottle choice MA intake (Wheeler et al., 2009; Shabani et al., 2011). Mice of each strain and sex were purchased from The Jackson Laboratory and reciprocally crossed to create the F1 generation, which were then reciprocally crossed to create the F2. Multiple replicate MADR lines have been serially created. For the current analysis, the 156 F2 mice that were selected as breeders for the first selection (S1) generations of the replicate 3, 4, and 5 MADR lines (half of each sex), based on the amount of MA consumed using the two-bottle choice MA drinking procedure described later, were genotyped for the *Taar1* polymorphism at position 229. Possible genotypes were homozygous *Taar1*^*m1J*^, homozygous *Taar1*^+^, and heterozygous for the two *Taar1* allele types. Mice were 53–68 days of age at the start of testing for MA intake (mean ± SEM = 59 ± 0.5 days).

### MADR mice

Five replicate sets of MAHDR and MALDR lines have been produced. *Taar1* position 229 SNP genotype and MA intake data for 208 MADR mice that served as breeders for various selection generations were available to examine genotype-phenotype association. Data were for mice selected as breeders from replicate 3 selection generation 3 (S3), replicate 4 S1, and replicate 5 S1 and S2. There were 26 mice (half of each sex) per selected line per replicate/generation. MA intake studies were initiated in these mice at 50–66 days of age (mean ± SEM = 59 ± 0.4 days).

### BXD RI mice

Breeding pairs for several BXD RI strains were obtained from RWW (University of Tennessee Health Science Center, Memphis, TN). Specific strains were chosen on the basis of their *Taar1* position 229 genotype (Shi et al., 2016) to allow tests of the associations between *Taar1* genotype and phenotypes. Breeders were established within the VAPORHCS and their offspring were tested for two-bottle choice MA intake, as described in Figure 1 and in our previous publications (Wheeler et al., 2009; Shabani et al., 2011). Mice from the following strains were tested and were homozygous for either the reference *Taar1*^+^ allele or the mutant *Taar1*^*m1J*^ allele: *Taar1*^+^ genotype: BXD154, BXD161, BXD184, BXD196, BXD199, BXD205, BXD212, and BXD215; mutant *Taar1*^*m1J*^ genotype: BXD113, BXD160, BXD171, BXD178, BXD186, BXD194, BXD204, and BXD210. The total number of mice tested for MA intake across all strains was 206 (120 female and 86 male), with a range of 3-28 per strain; mice were 53–114 days old (mean ± SEM = 79.2 ± 0.9). Some of the same BXD RI strains were also tested for MA-induced CTA and effects on core body temperature (independent sets of mice). For CTA, the strains were *Taar1*^+^ genotype: BXD154, BXD161, BXD196, BXD199, BXD205, and mutant *Taar1*^*m1J*^ genotype: BXD113, BXD178, and BXD216; for the temperature study, the strains were *Taar1*^+^ genotype: BXD154, BXD161, BXD184, BXD196, BXD199, BXD205 and BXD212, and mutant *Taar1*^*m1J*^ genotype: BXD113, BXD160, BXD171, BXD178, BXD194 and BXD210. The total number of mice tested across all strains for MA-induced CTA was 47 (24 female and 23 male) with a range of 1–7 per MA dose per strain, and mice were 72–98 days old (mean ± SEM = 83.2 ± 0.7 days). There were 223 BXD RI mice (117 female and 106 male) tested for MA effects on body temperature, with a range of 1–12 per MA dose per strain; mice were 49–124 days of age at the time of testing (mean ± SEM = 75 ± 0.9 days). The number of mice tested for some strains was small; however, to examine the genotype-phenotype associations, strain was not included as a factor; rather the BXD RI were considered as a single population. Thus, number tested per strain is informative, but not relevant to the current analysis, as all strains share a common C57BL/6J × DBA/2J background and the goal was to examine relationships with this particular genetic polymorphism.

### Standard inbred strains

DBA/2 mice were purchased from several vendors, including DBA/2J from The Jackson Laboratory, DBA/2NCrl from Charles River (Wilmington, MA, USA), DBA/2NHsd from Envigo (Indianapolis, IN, USA; formerly Harlan Sprague Dawley), and DBA/2NTac from Taconic (Albany, NY, USA). Six mice were obtained per sex per vendor and were genotyped for *Taar1* sequence at position 229 and tested for two-bottle choice MA consumption (Wheeler et al., 2009; Shabani et al., 2011). Mice were 56–70 days old at the initiation of testing (mean ± SEM = 56.7 ± 2.8 days). A separate cohort of mice from these four vendors (7–8 per sex per strain per dose) was tested for MA-induced CTA at age 73–93 days (mean ± SEM = 83.1 ± 1.1 days), and another cohort of DBA/2J and DBA/2NTac mice (11–12 per sex per strain per dose) was tested for MA-induced core temperature change at age 83–86 days (mean ± SEM = 84.5 ± 0.1 days); at the time of testing for thermal effects, DBA/2 mice were not available from some vendors.

### Drugs, reagents, and biological samples

(+)MA hydrochloride was purchased from Sigma (St. Louis, MO USA) and dissolved in tap water for drinking or in sterile 0.9% saline (Baxter Healthcare Corp., Deerfield, IL, USA) for injection. For CTA studies, sodium chloride (NaCl) was obtained from Sigma (St Louis, MO, USA) and 11.7 g of NaCl was dissolved in 1 liter of tap water to achieve a 0.2M solution. DNA samples used to narrow down the time when the spontaneous mutation arose were extracted from frozen spleen tissue archived at The Jackson Laboratory for DBA/2J mice from generations 208, 218, 221, and 225. These samples were sequenced locally for the *Taar1* SNP at position 229. Additional samples from generations 215 and 216 were sequenced by the technical division at The Jackson Laboratory, using our procedures (described below).

### *Taar1* sequencing

Genomic DNA was extracted from ear punch, tail, or spleen samples using QuickExtract DNA Extraction Solution (Epicenter, Madison, WI). *Taar1* was amplified using a Hotstart DNA polymerase kit (Qiagen, Valecia, CA), with sequence-specific primers surrounding the region of interest (see Harkness et al., 2015 for primer details). After amplification, PCR products were run on an agarose gel and then purified using a QIAquick gel extraction kit (Qiagen, Valencia, CA). Purified DNA samples were sequenced at the Oregon Health & Science University Sequencing Core using the forward and reverse primers to amplify the *Taar1* gene. Sequences of the PCR products were aligned with the mouse *Taar1* sequence (NM_053205.1). Some samples were genotyped using a rtPCR procedure that was more recently developed in our laboratory, simple to conduct and produces accurate sequence data, based on direct comparison of samples genotyped using both methods. Development of this rtPCR assay was based on standard Taqman methods (Shen et al., 2009) in which fluorescent probes were used to detect the SNP in the *Taar1* gene.

### Two-bottle choice MA drinking

Procedures were as described for selective breeding for two-bottle choice MA drinking (Wheeler et al., 2009; Shabani et al., 2011) and are summarized in Figure 1. Two initial days when mice had access to 2 tubes filled with tap water served to familiarize them with the novel drinking apparatus and provide total volume intake data prior to MA access. The 18-h per day MA access period has been used in all of our selection studies; pilot studies indicate that MA intake is higher with 6 h withdrawal between MA access periods, compared to 24-h access. The MA and water tube positions were switched every other day to account for potential drinking side bias. Consumption values for the second and fourth days of access for each MA concentration are averaged for each mouse, because these days represent the second day after a tube position switch, when mice should be fully familiar with the location of the MA tube. MA consumption was measured in ml (accuracy = 0.2 ml) and then converted to mg/kg, based on body weight measured every 2 days. Total volume consumed (ml) prior to MA access and during the 18-h MA access periods were also analyzed.

### MA-induced CTA

Mice were tested for sensitivity to the conditioned aversive effect of 2 mg/kg MA, using established procedures that utilize a 0.2M NaCl solution as a conditioned cue for the interoceptive effects of MA (Table 2). Mice with a low level of MA intake exhibit profound CTA to this dose of MA, indicated by a reduction in NaCl consumption (ml) across MA treatment days, whereas mice with a high level of MA intake exhibit no CTA to this dose (Wheeler et al., 2009; Shabani et al., 2011; Harkness et al., 2015). Mice were singly-housed, acclimated for 2 days to drinking from 10-ml graduated cylinders fitted with stoppers and sipper tubes (study days −1 and 0), and then their water access was limited to 2 h per day for a 4-day acclimation period to induce motivation to drink the novel NaCl solution (study days 1–4). Beginning on day 5, the NaCl solution was offered for 1 h every other day for a total of 6 presentations (days 5, 7, 9, 11, 13, and 15), and consumption was measured in ml by measuring tube volumes before and after each drinking session. Except on day 5 (when mice were introduced to the novel taste to reduce neophobia) and 15 (the last test day), mice were injected with saline or 2 mg/kg MA immediately after the 1-h NaCl drinking period. Day 5 data were not included in the analysis for CTA, consistent with our previous studies (Wheeler et al., 2009; Shabani et al., 2011; Harkness et al., 2015). About 3 h post-injection, mice were given access to water for 30 min each day to avoid dehydration. On intervening days, mice had 2 h of access to water and there were no experimental manipulations.

**Table 2:** Protocol for measuring MA-induced conditioned taste aversion (CTA).

**Group**	**Day−1-0**	**Day 1–4**	**Day 5**	**Day 6, 8, 10, 12, 14**	**Day 7, 9, 11, 13**	**Day 15**
Saline	Acclimation to drinking tubes	Water restricted to 2 h/day	1 h NaCl; No inj.; 30 min water	2 h/day water	1 h NaCl; Saline inj.; 30 min water	1 h NaCl; No inj.; end of study
2 mg/kg MA	Acclimation to drinking tubes	Water restricted to 2 h/day	1 h NaCl; No inj.; 30 min water	2 h/day water	1 h NaCl; MA inj.; 30 min water	1 h NaCl; No inj.; end of study

### MA effects on core body temperature

Mice were tested for the effect of 2 mg/kg MA on body temperature at an ambient temperature of 21 ± 2°C. This dose of MA was chosen based on previous results indicating that mice with a low level of MA intake exhibit a profound hypothermic response to 2 mg/kg MA at this ambient temperature that is absent in mice with a higher level of MA intake (Harkness et al., 2015). Mice were weighed and then placed into individual perforated acrylic plastic cubicles (8 × 19 × 8 cm in W × H × L), that served to prevent huddling-associated alterations in body temperature (Crabbe et al., 1987, 1989). After placement, they remained undisturbed for 1 h and then a baseline rectal temperature was obtained (T0). Mice then received an injection of saline or MA, were returned to their respective cubicles, and rectal temperature was again measured at 30, 60, 90, 120, and 180 min post-injection (T30-180). This time-course was determined from our former research results (Harkness et al., 2015). To obtain these temperatures, each mouse was removed from its cubicle, gently restrained by the scruff of the neck, and a glycerin-coated, 5 mm probe attached to a Thermalert model TH-8 digital thermometer (Sensortek, Clifton, NJ) was inserted 2.5 cm into the rectum for 5 s. The mouse was then immediately returned to its cubicle.

### Data analysis

Statistica 64 Academic Software (Dell, Inc., Tulsa, OK, USA) was used for all analyses. Animals of both sexes were used in all experiments. MA intake (mg/kg), total volume consumed (ml), and NaCl volume intake (ml) data (for CTA studies) were analyzed by repeated measures analysis of variance (ANOVA) with MA concentration or test day as the repeated measure, and sex, genotype or vendor, replicate/generation, and dose as possible independent variables. MA-induced changes in body temperature were analyzed by repeated measures ANOVA with time as the repeated measure, and sex, genotype or vendor, and dose as possible independent variables. Effects were considered statistically significant at *p* < 0.05. Two-way interactions were interpreted using simple main effects analysis and *post hoc* Newman-Keuls mean comparisons were performed when appropriate. Pearson's r was used to calculate correlations between phenotypes and *Taar1* genotype. The chi-squared test was used to compare expected vs. observed *Taar1* genotype frequencies.

## Results

### Frequency of *Taar1* genotypes in F2 and MADR mice

The key findings from this analysis are that there was a higher frequency of the homozygous *Taar1*^*m1J*^ genotype in populations of mice chosen to serve as breeders for the high MA intake lines, compared to the low MA intake lines, and that this difference arose as early as in the F2 mice that served as breeders for the first selection generation.

### F2 mice

**Figure 2:**
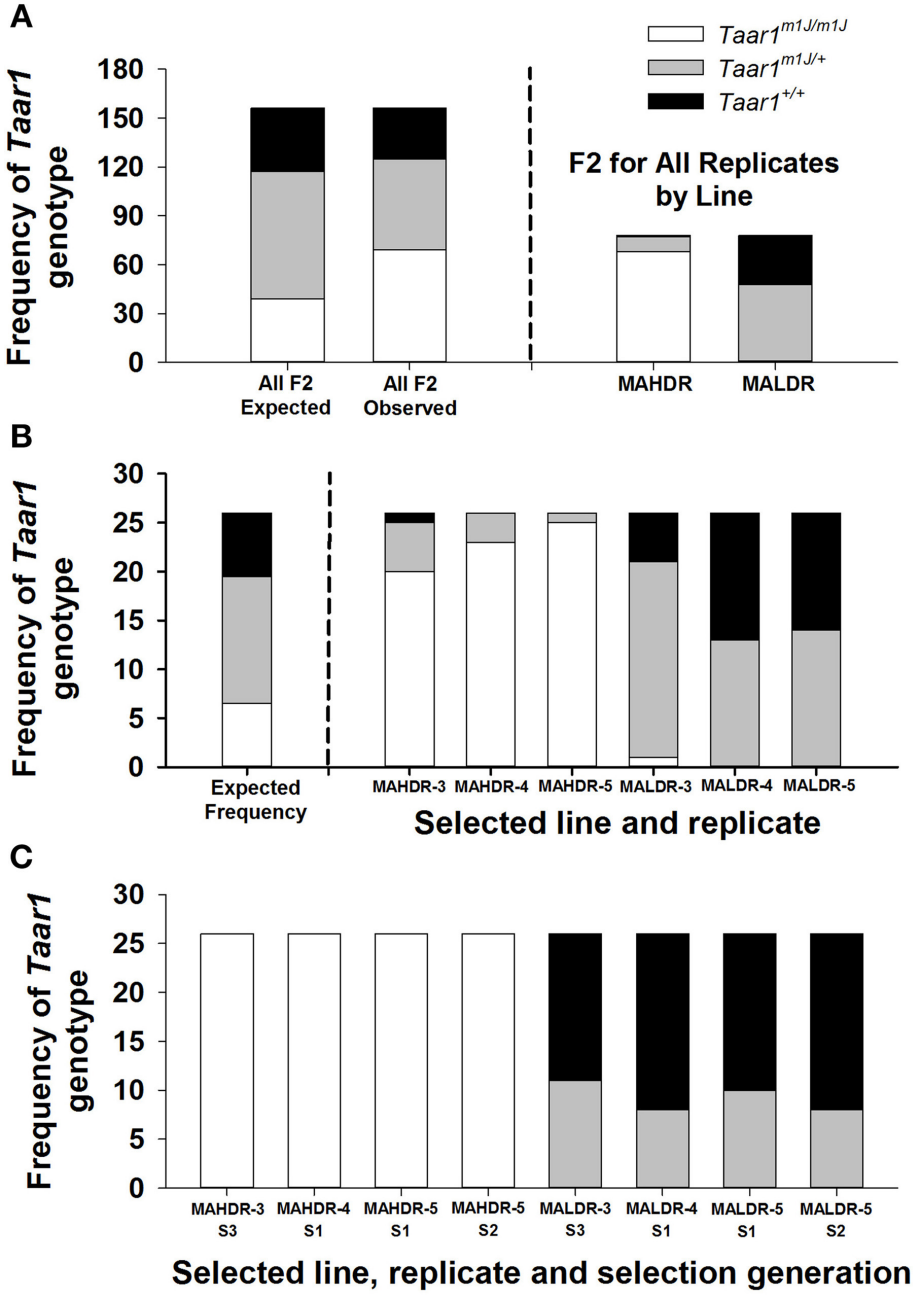
*Taar1* genotype frequencies are altered by selective breeding for high and low levels of MA intake, beginning with a founding population of C57BL/6J × DBA/2J F2 mice. Shown in **A,B** are the frequencies of *Taar1* genotypes for F2 mice that were tested for MA drinking and selected to produce the first selection generation (S1) offspring of several replicates of the MADR lines of mice. **(A)** Expected frequency (based on the null hypothesis of no role for *Taar1* in MA intake) and observed frequency of homozygous *Taar1*^*m1J*^ (in white), heterozygous *Taar1*^*m*1*J*/+^ (in gray), and homozygous *Taar1*^+^ (in black) mice among all selected F2 breeders (left) and observed frequencies for the F2 breeders separated by selected line (right). **(B)** Expected *Taar1* genotype frequency (left) and observed frequencies for each line and replicate (right). **(C)** *Taar1* genotype frequencies for the breeders of several replicates and selection generations (Sx) of the MAHDR and MALDR lines.

*Taar1* genotype results for the 156 F2 mice that were tested for MA intake and selected to produce the S1 generations of the replicate 3, 4, and 5 MADR lines are illustrated in Figure 2. In Figure 2A (left panel) are the expected genotype frequencies for the null hypothesis that *Taar1* genotype is not relevant to MA intake, alongside the actual frequencies of *Taar1* genotypes. Also in Figure 2A (right panel) are genotype frequencies for the F2 mice selected as breeders specifically for the MAHDR vs. MALDR lines (replicates combined). The expected 1:2:1 ratio of *Taar1*^m1J/m1J^: *Taar1*^m1J/+^: *Taar1*^+/+^ genotypes for the null hypothesis (which is 39:78:39 for 156 mice) significantly diverged from the observed ratio of 31:56:69 (chi-squared = 30.9, *p* < 0.001; Figure 2A, left panel). The larger number of *Taar1*^*m1J*^ homozygotes among the F2 chosen as breeders of the MAHDR lines, compared to MALDR lines, is apparent (68:1; Figure 2A, right panel). In Figure 2B are the expected genotype frequencies for the null hypothesis (which is 1:2:1 for each line or 6.5:13:6.5) and the observed genotype frequencies for each replicate, to illustrate consistency of the results. Again, the genotype frequencies for each population of F2 mice chosen to serve as breeders, based on their extreme high or low MA drinking phenotypes, significantly diverged from the expected frequencies. Chi-squared values were 161.3 (*p* < 0.001) and 24.8 (*p* < 0.001) for MAHDR and MALDR collapsed on replicate, respectively. The larger frequency of the *Taar1*^m1J/m1J^ genotype in each MAHDR line, compared to each MALDR line is apparent; 20:1, 23:0 and 25:0 for replicate 3, 4, and 5, respectively.

### MADR mice

Data from the F2 populations indicate rapid fixation of the *Taar1*^*m1J*^ allele in the MAHDR lines. However, whether fixation of the *Taar1*^+^ allele was achieved or heterozygosity remained in the MALDR lines by the end of selection was unknown. To examine this, we searched our archives for animals from the various replicate sets of lines and generations for which we had both *Taar1* genotype and MA intake data. The overall outcome of this analysis is that heterozygosity, rather than fixation of the *Taar1*^+^ allele, was retained in the MALDR lines at the end of selection. *Taar1* genotype frequencies are shown in Figure 2C for four sets of animals. Genotyping results identified all MAHDR mice (*n* = 104) as homozygous *Taar1*^*m1J*^. This is consistent with the high frequency of *Taar1*^*m1J*^ homozygotes that were selected from the F2 population to generate MAHDR lines of multiple replicates (Figure 2B). The MALDR mice were all heterozygous or homozygous *Taar1*^+^, again consistent with findings for the F2 mice that served as breeders of the MALDR lines (Figure 2B). When considering all 104 MALDR mice, the observed ratio of *Taar1*^m1J/+^*: Taar1*^+/+^ was 37:67. Assuming an equal probability of either genotype serving as a member of the breeding pairs for the MALDR lines, the expected ratio would be 1:4:7 *Taar1*^m1J/m1J^: *Taar1*^m1J/+^: *Taar1*^+/+^, since possible breeding combinations would be *Taar1*^+/+^ x *Taar1*^+/+^; *Taar1*^m1J/+^ x *Taar1*^+/+^, and *Taar1*^m1J/+^ x *Taar1*^m1J/+^. Thus, one animal in twelve would be expected to be a *Taar1*^*m1J*^ homozygote, to exhibit higher MA intake, and be unlikely to be subsequently selected as a breeder. If all breeders are binned into the *Taar1*^m1J/+^ and *Taar1*^+/+^ classes, and the *Taar1*^m1J/m1J^ animals are “lost,” the predicted ratio of *Taar1*^m1J/+^*: Taar1*^+/+^ would be 4:7, which is 38:66 for the current 104 MALDR breeders. The observed ratio of these genotypes was 34:67, which did not significantly differ from expectation, based on the chi-squared test. Frequencies by replicate/generation (Figure 2C) did not significantly differ from the expected 4:7 ratio (9.5:16.5 for the 26 animals per generation). Furthermore, there was little change in the ratio with increasing selection generation. The remaining heterozygosity is consistent with dominance of the *Taar1*^+^ allele for level of MA intake.

### MA drinking in F2 and MADR mice

The key finding from this analysis in both the F2 and MADR mice is that *Taar1* genotype is strongly associated with MA intake.

### F2 mice

**Figure 3:**
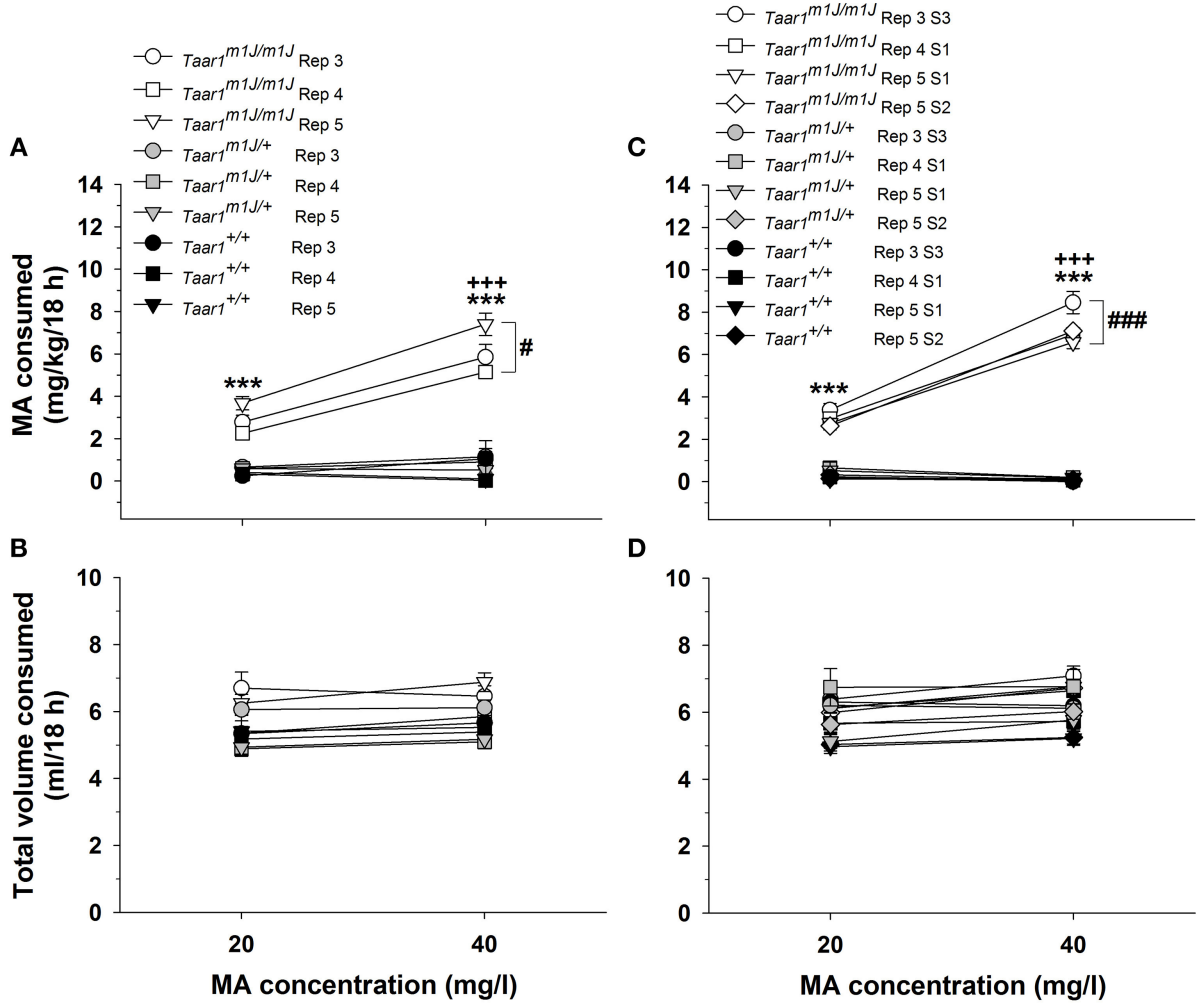
The homozygous *Taar1*^*m1J*^ genotype is associated with higher MA intake. **(A)** Mean ± SEM MA consumption when MA was offered vs. water in 20 and 40 mg/l concentrations for F2 mice selected to produce the S1 generation MADR line offspring of replicate (Rep) 3–5, separated by *Taar1* genotype; *Taar1* genotype frequencies for these mice are those in Figures 2A,B. **(B)** Mean ± SEM total amount of fluid consumed by the same F2 mice from both the water and MA tubes, during the 18-h period when MA was available. **(C)** Mean ± SEM MA consumption when MA was offered vs. water in 20 and 40 mg/l concentrations to MADR mice of several replicates and selection generations, for which *Taar1* genotype frequencies are shown in Figure 2C. **(D)** Mean ± SEM total amount of fluid consumed by the same MADR mice from both the water and MA tubes, during the 18-h period when MA was available. ^***^p < 0.001 for the comparison of homozygous *Taar1*^*m1J*^ to heterozygous and homozygous *Taar1*^+^ mice; +++*p* < 0.001 for the comparison of MA consumption from the 20 and 40 mg/l MA concentrations within the homozygous *Taar1*^*m1J*^ mice; ^#^*p* < 0.05 for overall more MA consumed in Rep 5 compared to Rep 3 and Rep 4 homozygous *Taar1*^*m1J*^ mice; ###*p* < 0.001 for overall more MA consumed in Rep 3 S3 *Taar1*^*m1J*^ mice, compared to Rep 4 S1 and Rep 5 S1 and S2 *Taar1*^*m1J*^ mice.

MA consumption data for the F2 mice are presented in Figure 3A. A repeated measures ANOVA was performed on MA intake data, with sex, *Taar1* genotype, replicate, and MA concentration as independent variables. There were no significant main or interaction effects involving sex, so data were further analyzed for the sexes combined. There was a significant *Taar1* genotype × MA concentration interaction [*F*_(2, 147)_ = 64.4, *p* < 0.0001]; homozygous *Taar1*^*m1J*^ mice consumed significantly more MA than *Taar1*^m1J/+^ or homozygous *Taar1*^+^ mice for both MA concentrations, and consumed more MA when the concentration was 40 mg/l. Heterozygous *Taar1*^m1J/+^ mice and homozygous *Taar1*^+^ mice did not differ from each other or exhibit MA concentration-dependent effects. There was a significant *Taar1* genotype × replicate interaction [*F*_(4, 147)_ = 3.9, *p* < 0.01] that was associated with higher MA intake in homozygous *Taar1*^*m1J*^ replicate 5 F2 mice, compared to homozygous *Taar1*^*m1J*^ F2 mice from replicates 3 and 4.

Total amount of fluid consumed during the time that MA was available was also impacted by *Taar1* genotype in the F2 mice (Figure 3B). There were no significant effects involving sex in the initial analysis, so data were considered for the sexes combined. There were significant main effects of *Taar1* genotype [*F*_(2, 147)_ = 11.0, *p* < 0.0001], MA concentration [*F*_(1, 147)_ = 8.3, *p* < 0.01] and replicate [*F*_(2, 147)_ = 4.9, *p* < 0.01], but no significant interaction effects. Homozygous *Taar1*^*m1J*^ mice consumed more total volume than *Taar1*^m1J/+^ or homozygous *Taar1*^+^ mice (mean ± SEM = 6.2 ± 0.1 vs. 5.4 ± 0.2 and 5.4 ± 0.2 ml, respectively; *p*s < 0.01), total volume consumed was greater when the 40 mg/l MA concentration was available (mean ± SEM = 5.6 ± 0.1 vs. 5.8 ± 0.1 ml for 20 and 40 mg/l, respectively; *p* < 0.01), and replicate 4 mice consumed less total volume than replicates 3 and 5 (mean ± SEM = 6.1 ± 0.2, 5.3 ± 0.2 and 5.7 ± 0.2 ml for replicate 3, 4, and 5, respectively; *p*s < 0.05). When water intake data for the same 18-h period averaged for the 2 days prior to MA access were similarly analyzed, there was no significant sex, *Taar1* genotype, or replicate effect.

Finally, correlations were calculated between *Taar1* genotype and intake measures. Results are presented in Table 3. First, correlations were considered for mice of the 3 possible *Taar1* genotypes: *Taar1*^m1J/m1J^, *Taar1*^m1J/+^, and *Taar1*^+/+^. For MA intake, the correlations with MA intake were highly significant and *Taar1* genotype accounted for 46 and 55% of the variance in MA intake for the 20 and 40 mg/l MA concentrations, respectively. For total volume, the correlations were smaller, but also significant, with *Taar1* genotype accounting for 6 and 10% of the variance, during the periods when the 2 MA concentrations were available. Data were also examined with mice that were *Taar1*^m1J/+^, and *Taar1*^+/+^ coded as a single class, based on evidence for dominance of the *Taar1*^+^ allele on MA intake (Harkness et al., 2015). The amount of variance for which *Taar1* genotype accounted increased to 55 and 66% for MA intake for the 20 and 40 mg/l concentrations, respectively (Table 3). Recoding the data in this way had a smaller impact on the amount of variance in total volume consumed accounted for by *Taar1* genotype, with values of 6 and 12%.

**Table 3:** *Taar1* genotype-phenotype correlations for multiple genetic models.

	**F2 coded as 3 *Taar1* genotypes**	**F2 coded as 2 *Taar1* genotypes**	**MADR coded as 3 *Taar1* genotypes**	**MADR coded as 2 *Taar1* genotypes**	**BXD RI mice**	**DBA/2 mice**
MA intake (mg/kg) 20 mg/l	***r*** = **0.68** *p* < 0.00001 (*N* = 156)	***r*** = **0.74** *p* < 0.00001 (*N* = 156)	***r*** = **0.78** *p* < 0.00001 (*N* = 208)	***r*** = **0.82** *p* < 0.00001 (*N* = 208)	***r*** = **0.73** *p* < 0.00001 (*N* = 206)	***r*** = **0.85** *p* < 0.00001 (*N* = 48)
MA intake (mg/kg) 40 mg/l	***r*** = **0.74** *p* < 0.00001 (*N* = 156)	***r*** = **0.81** *p* < 0.00001 (*N* = 156)	***r*** = **0.87** *p* < 0.00001 (*N* = 208)	***r*** = **0.94** *p* < 0.00001 (*N* = 208)	***r*** = **0.82** *p* < 0.00001 (*N* = 206)	***r*** = **0.96** *p* < 0.00001 (*N* = 48)
Total volume (ml) 20 mg/l	***r*** = **0.24** *p* < 0.01 (*N* = 156)	***r*** = **0.25** *p* < 0.01 (*N* = 156)	***r*** = **0.25** *p* < 0.001 (*N* = 208)	***r*** = **0.22** *p* < 0.005 (*N* = 208)	*r* = 0.13 *p* = 0.063 (*N* = 206)	*r* = −0.24 *p* = 0.100 (*N* = 48)
Total volume (ml) 40 mg/l	***r*** = **0.31** *p* < 0.0001 (*N* = 156)	***r*** = **0.34** *p* < 0.0001 (*N* = 156)	***r*** = **0.44** *p* < 0.00001 (*N* = 208)	***r*** = **0.41** *p* < 0.00001 (*N* = 208)	*r* = 0.12 *p* = 0.086 (*N* = 206)	*r* = −0.15 *p* = 0.309 (*N* = 48)
CTA: NaCl consumption 2 mg/kg MA	No correlation data	No correlation data	No correlation data	No correlation data^*^	***r*** = **0.96** *p* < 0.00001 (*N* = 20)	***r*** = **0.83** *p* < 0.00001 (*N* = 32)
CTA: NaCl consumption saline group	No correlation data	No correlation data	No correlation data	No correlation data^*^	*r* = −0.24 *p* = 0.228 (*N* = 27)	*r* = 0.13 *p* = 0.486 (*N* = 31)
Thermal response 2 mg/kg MA	No correlation data	No correlation data	No correlation data	No correlation data^*^	***r*** = **0.71** *p* < 0.00001 (*N* = 171)	***r*** = **0.82** *p* < 0.00001 (*N* = 47)
Thermal response saline group	No correlation data	No correlation data	No correlation data	No correlation data^*^	*r* = 0.03 *p* = 0.712 (*N* = 154)	*r* = 0.10 *p* = 0.499 (*N* = 48)

Bolded r values indicate statistical significance.

* *Although individual animal Taar1 genotype-phenotype correlations were not performed for these traits, published MAHDR vs. MALDR line differences reflect largely homozygous Taar1^m1J^ for MAHDR vs. data for heterozygotes and homozygous Taar1^+^ for MALDR, based on genotype frequency information given in Figure 2. Thus, the homozygous Taar1^m1J^ genotype is associated with higher NaCl consumption across days (so reduced CTA) and reduced MA-induced hypothermia in the MADR mice (Wheeler et al., 2009; Shabani et al., 2011, 2012; Harkness et al., 2015)*.

### MADR mice

A repeated measures ANOVA was performed on MA intake data (Figure 3C), with sex, *Taar1* genotype, replicate/generation and MA concentration as independent variables. There was a significant *Taar1* genotype x MA concentration interaction [*F*_(2, 183)_ = 272.9, *p* < 0.0001], but genotype-dependent effects were significant for both the 20 and 40 mg/l MA concentrations (*p*s < 0.001). Homozygous *Taar1*^*m1J*^ mice consumed significantly more MA than heterozygotes and homozygous *Taar1*^+^ mice for both MA concentrations. MA intake was concentration-dependent only in homozygous *Taar1*^*m1J*^ mice; they consumed more MA when the 40 mg/l concentration was available. There was also a significant *Taar1* genotype × replicate/generation interaction [*F*_(6, 183)_ = 4.0, *p* < 0.001]. The S3 homozygous *Taar1*^*m1J*^ mice from replicate 3 consumed more MA compared to the S1 and S2 *Taar1*^*m1J*^ homozygotes from the other replicates (mean ± SEM = 5.9 ± 0.15 vs. 5.0 ±.15, 4.7 ± 0.15, and 4.9 ± 0.15 mg/kg for replicate 3 S3 vs. replicate 4 S1, replicate 5 S1 and replicate 5 S2, respectively; *p*s < 0.001), supporting an effect of continued selection for high MA intake. There were no significant differences between the heterozygote and *Taar1*^+^ homozygote mice, within or across replicate/generation, indicating rapid fixation of the low MA intake trait in breeders chosen for selection. Finally, there was a significant sex × *Taar1* genotype interaction [*F*_(2, 183)_ = 7.7, *p* < 0.001]. Female, homozygous *Taar1*^*m1J*^ mice consumed significantly more MA than male, homozygous *Taar1*^*m1J*^ mice (mean ± SEM = 5.5 ± 0.1 and 4.7 ± 0.1 mg/kg, respectively; *p* < 0.001), whereas there were no sex differences within the heterozygote or *Taar1*^+^ homozygote genotypes.

There were no significant effects involving sex for total amount of fluid consumed during the time that MA was available, so data were considered for the sexes combined (Figure 3D). There was a significant main effect of replicate/generation [*F*_(3, 195)_ = 5.1, *p* < 0.01]; total volume for S1 and S2 replicate 5 animals was less than for replicate 3 S3 mice (mean ± SEM = 5.7 ±.16 and 5.8 ±.16 vs. 6.4 ±.16 ml, respectively; *p*s < 0.01). There was also a significant *Taar1* genotype x MA concentration interaction [*F*_(2, 195)_ = 11.7, *p* < 0.0001]. Homozygous *Taar1*^*m1J*^ mice consumed more total volume than homozygous *Taar1*^+^ mice, for both MA concentrations (mean ± SEM = 6.2 ±.11 vs. 5.5 ±.14 and 6.8 ±.11 vs. 5.6 ±.14 ml, respectively; *p*s < 0.01). Homozygous *Taar1*^*m1J*^ mice consumed more total volume than mice heterozygous for *Taar1* allele type only when the 40 mg/l MA concentration was offered (mean ± SEM = 6.8 ±.11 vs. 6.2 ±.19 ml, respectively; *p* < 0.01). Also, during the time that the 40 mg/l MA concentration was offered, mice heterozygous for the *Taar1* allele consumed significantly more total volume than *Taar1*^+^ mice (mean ± SEM = 6.2 ±.19 vs. 5.6 ±.14 ml, respectively; *p* < 0.05). When water intake data for the same 18-h period averaged for the 2 days prior to MA access were similarly analyzed, there were no significant sex or *Taar1* genotype effects.

Finally, correlations were calculated between *Taar1* genotype and intake measures during the MA access period. Results are presented in Table 3. When data were coded as the 3 possible genotypes, *Taar1* genotype accounted for 61 and 76% of the variance in MA intake for the 20 and 40 mg/l MA concentrations, respectively. For total volume, the correlations were smaller, but also significant, with *Taar1* genotype accounting for 6 and 19% of the variance during the periods when the 2 MA concentrations were available. When data for the heterozygotes and *Taar1*^+^ homozygotes were coded as a single group, the amount of variance for which *Taar1* genotype accounted increased to 67 and 88% for MA intake for the 20 and 40 mg/l MA concentrations, respectively. Recoding the data in this way had little impact for total volume consumed, with *Taar1* genotype accounting for 5 and 17% of the variance.

### *Taar1* genotype and MA intake in BXD RI mice

**Figure 4:**
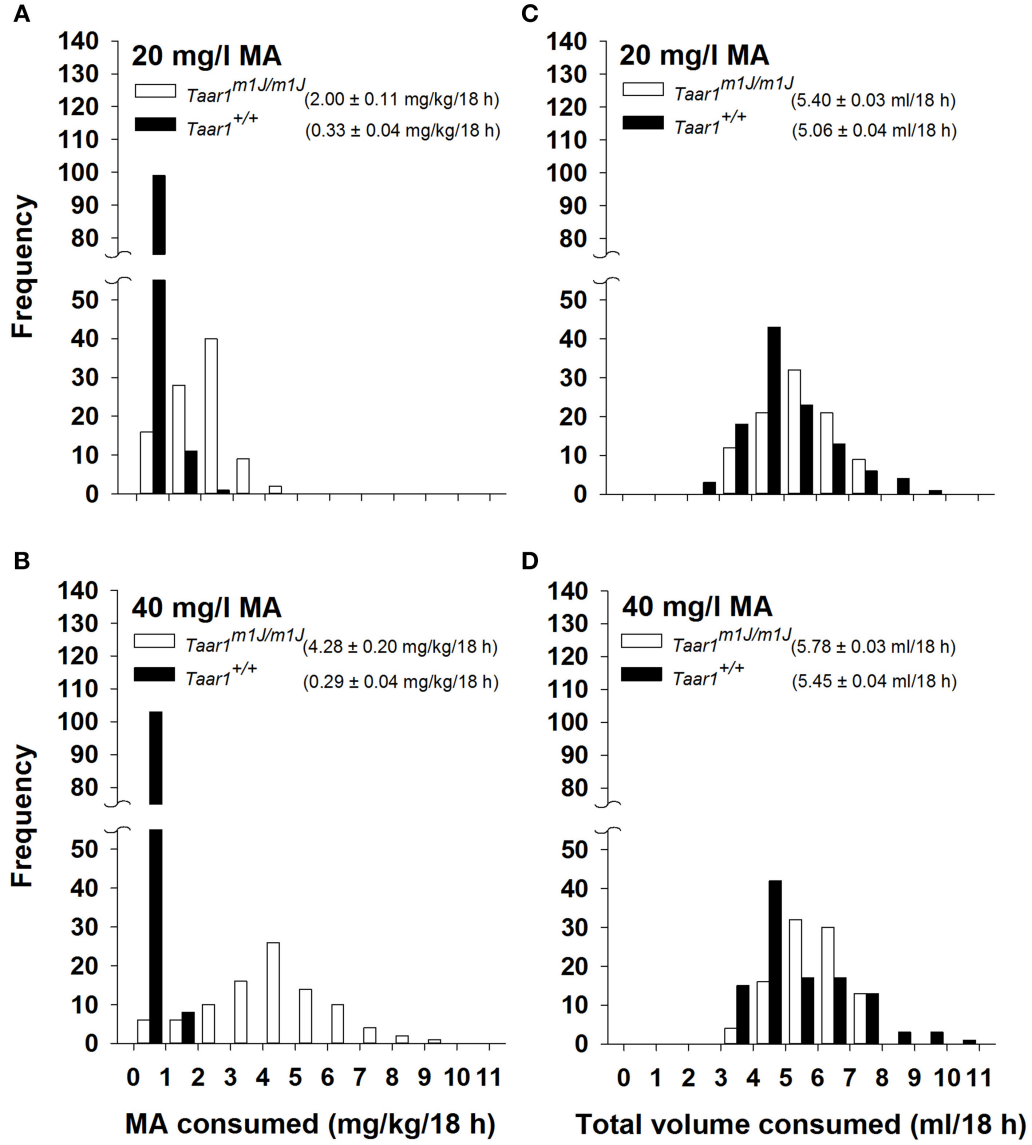
Frequency distributions for **(A)** 20 mg/l MA consumed, **(B)** 40 mg/l MA consumed, **(C)** total volume consumed during 20 mg/l MA access, and **(D)** total volume consumed during 40 mg/l MA access in 206 BXD RI mice, according to *Taar1* genotype. Mean ± SEM MA or total volume consumed is given in the legend for each *Taar1* genotype.

Data plotted in Figures 4A,B illustrate the frequency of the 2 possible *Taar1* genotypes with regard to amount of MA consumed for the 206 BXD RI mice, and confirm the association of homozygosity for *Taar1*^*m1J*^ with heightened MA intake. Because these mice are inbred, all individuals were either homozygous *Taar1*^*m1J*^ (*n* = 95; 53 male and 42 female) or homozygous *Taar1*^+^ (*n* = 111; 67 male and 44 female). MA intake was impacted by *Taar1* genotype. A repeated measures ANOVA with MA consumption data grouped on sex, *Taar1* genotype, and MA concentration identified a significant *Taar1* genotype x MA concentration interaction [*F*_(1, 202)_ = 212.0, *p* < 0.0001], with homozygous *Taar1*^*m1J*^ mice consuming significantly more MA from both the 20 (Figure 4A) and 40 (Figure 4B) mg/l concentration solutions, compared to homozygous *Taar1*^+^ mice (*p*s < 0.0001). Only the *Taar1*^*m1J*^ homozygotes exhibited concentration-dependent MA intake and consumed significantly more MA when the MA concentration was 40 mg/l, compared to 20 mg/l (*p* < 0.0001). There was also a significant sex × *Taar1* genotype interaction [*F*_(2, 202)_ = 4.0, *p* < 0.05]. Female, homozygous *Taar1*^*m1J*^ mice consumed about 0.5 mg/kg more MA compared to males (mean ± SEM = 3.4 ± 0.13 vs. 2.9 ± 0.15 mg/kg, respectively; *p* < 0.05); there was no sex difference in homozygous *Taar1*^+^ mice.

*Taar1* genotype frequency data are plotted for total volume consumed in Figures 4C,D. There were no effects of sex for total volume and *Taar1* genotype did not have a significant impact on total volume consumed. However, there was a significant effect of MA concentration, with about 0.39 ml more total volume consumed during the period when the 40 mg/l MA concentration was available [*F*_(1, 202)_ = 65.7, *p* < 0.0001]. When water intake data for the same 18-h period averaged for the 2 days prior to MA access were similarly analyzed, there were no significant sex or *Taar1* genotype effects. *Taar1* genotype and total fluid intake were not significantly correlated (Table 3), whereas *Taar1* genotype explained 53 and 67% of the variance in MA intake for the 2 MA concentrations, respectively, in the BXD RI mice.

### *Taar1* genotype and MA intake in DBA/2 mice

**Figure 5:**
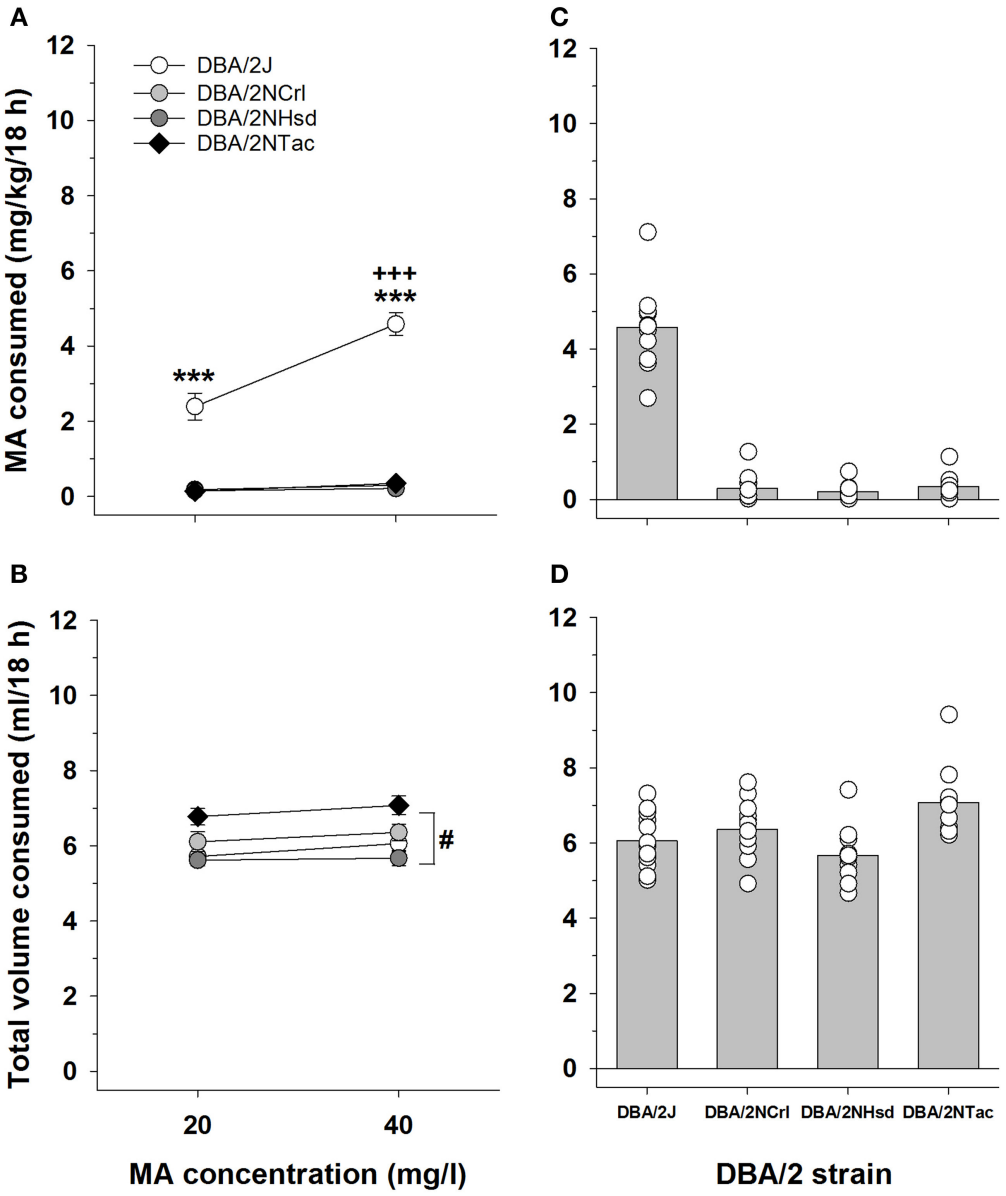
MA and total volume consumed differ across DBA/2 mice sourced from four vendors. **(A)** Mean ± SEM MA consumed when MA was offered vs. water in 20 and 40 mg/l concentrations to DBA/2 mice from The Jackson Laboratory (DBA/2J), Charles River (DBA/2NCrl), Envigo (DBA/2NHsd), and Taconic (DBA/2NTac). **(B)** Mean ± SEM total volume of fluid consumed from both the water and MA tubes, during the 18-h period when MA was available. **(C)** Means and individual MA intake amounts for the 40 mg/l MA concentration. **(D)** Means and individual total volumes consumed during the 18-h period when the 40 mg/l MA concentration was offered. ^***^p < 0.001 for the comparison of DBA/2J to mice from all other vendors; ^+++^*p* < 0.001 for comparison of MA consumed from the 20 and 40 mg/l MA concentrations within the DBA/2J mice; ^#^*p* < 0.05 for overall more total volume consumed in DBA/2NTac mice, compared to mice from all other vendors.

The DBA/2 mice sourced from different vendors provide a more isogenic background on which to examine the relation between *Taar1* genotype and MA intake. The key findings from this study are that *Taar1* genotype differed in DBA/2 mice sourced from different vendors, all DBA/2 mice from The Jackson Laboratory were homozygous for the *Taar1*^*m1J*^ allele, all DBA/2 mice from other vendors were homozygous for the *Taar1*^+^ allele, and homozygosity for the *Taar1*^*m1J*^ allele was associated with greater MA intake. A repeated measures ANOVA was performed on MA intake (Figure 5A) with sex, vendor and MA concentration as independent variables. There were no significant main or interaction effects involving sex, so data were further analyzed for the sexes combined. There was a significant vendor x MA concentration interaction [*F*_(3, 44)_ = 67.4; *p* < 0.0001]. Only the DBA/2J mice exhibited concentration-dependent MA intake, and there was a significant vendor effect for both MA concentrations (*p*s < 0.001). The DBA/2J mice had higher MA intake than DBA/2 mice from all other vendors at both the 20 and 40 mg/l concentrations, whereas there were no significant differences in MA intake among the DBA/2NCrl, DBA/2NTac, and DBA/2NHsd mice.

For total volume of fluid consumed (Figure 5B), there were no significant effects of sex in the initial analysis so data were considered for the sexes combined. There was a significant main effect of vendor [*F*_(3, 44)_ = 8.0, *p* < 0.001], with the DBA/2NTac mice consuming significantly more fluid than the DBA/2 mice from other vendors (*p*s < 0.05), and comparable total volumes for the DBA/2J, DBA/2NCrl, and DBA/2NHsd mice. There was also a main effect of concentration [*F*_(1, 44)_ = 9.8, *p* < 0.01], with about 0.25 ml more total volume consumed during the period when the 40 mg/l MA concentration was available. When water intake data for the same 18-h period averaged for the 2 days prior to MA access were similarly analyzed, there was a significant effect of vendor [*F*_(3, 40)_ = 6.0, *p* < 0.01]. DBA/2NCrl mice consumed more water than DBA/2NhSD and DBA/2J mice, but not DBA/2NTac, which consumed more than DBA/2J.

Individual MA and total fluid intake values for the 40 mg/l MA concentration are plotted in Figures 5C,D to better illustrate the range and distribution of individual differences. There was a wider range of MA intake values for DBA/2J mice, compared to mice from the other vendors; however, there was no overlap of individual values for the DBA/2J animals with values for the other DBA/2 mice. In contrast, there was considerable overlap of individual values across all vendors for total volume. *Taar1* genotype explained 72 and 92% of the variance in MA intake for the 20 and 40 mg/l MA concentrations, respectively, in the DBA/2 mice. The correlations between *Taar1* genotype and total fluid intake were not significant (Table 3).

### *Taar1* genotype and MA-induced CTA

The main finding for the CTA studies is that *Taar1* genotype was associated with sensitivity to MA-induced CTA, with homozygous *Taar1*^*m1J*^ mice exhibiting resistance.

### BXD RI mice

**Figure 6:**
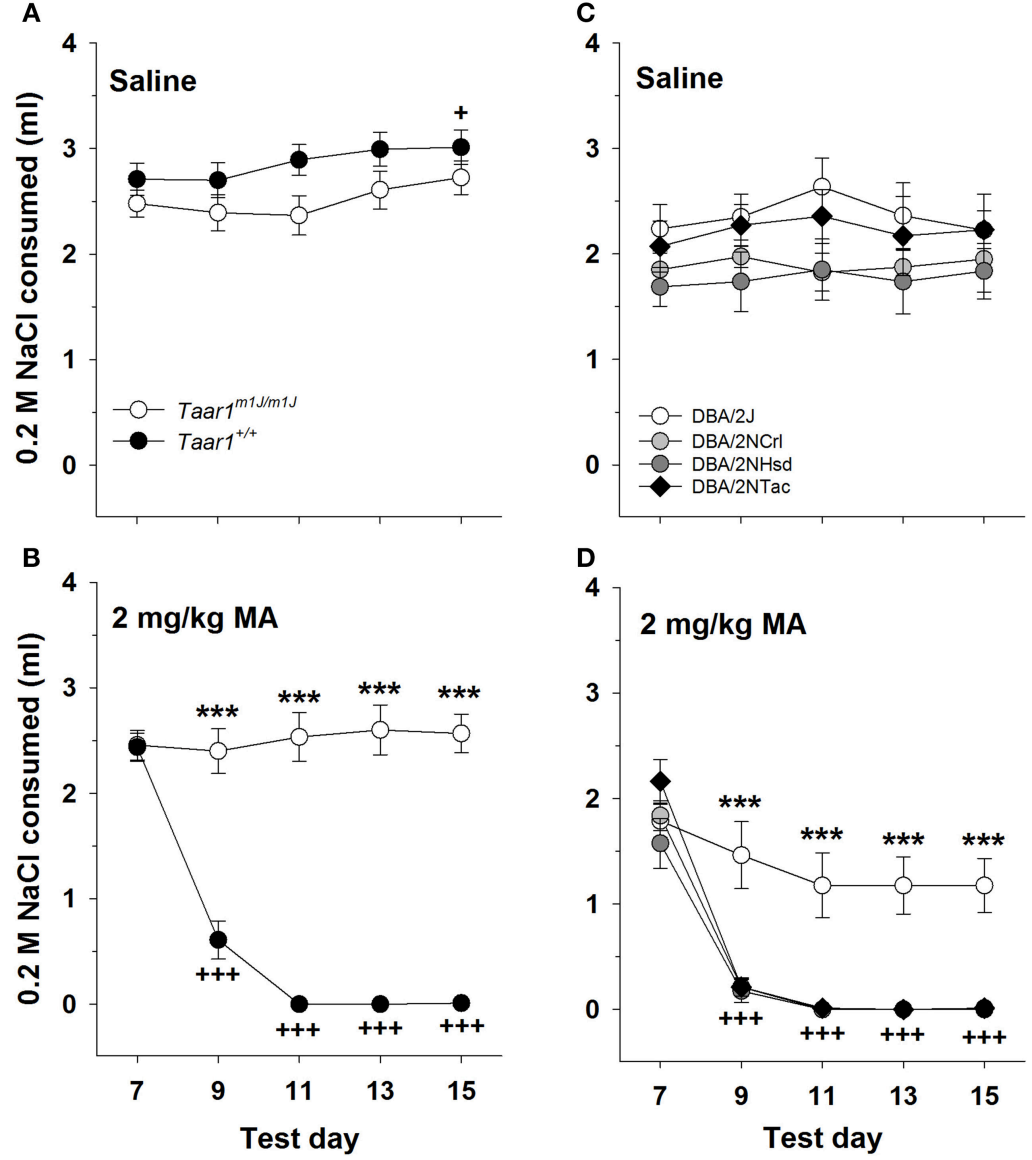
The *Taar1*^*m1J/m1J*^ genotype is associated with insensitivity to MA-induced CTA. Mean ± SEM NaCl consumed initially (Test day 7) and after pairing with saline **(A)** or 2 mg/kg MA **(B)** injections (Test days 9–15) in homozygous *Taar1*^*m1J*^ *and Taar1*^+^ BXD RI mice. For panels **(A,B)** ^***^p < 0.001 for the comparison of the two genotypes on the indicated test day; ^+^*p* < 0.05 for overall NaCl intake on test day 15, compared to test day 9; ^+++^*p* < 0.001 for NaCl intake in *Taar1*^+/+^ mice on each indicated day, compared to test day 7. Mean ± SEM NaCl consumed initially (Test day 7) and after pairing with saline **(C)** or 2 mg/kg MA **(D)** injections (Test days 9–15) in DBA/2 mice. For panels **(C,D)** ^***^p < 0.001 for the comparison of DBA/2J mice to mice of the other 3 vendors on the indicated test day; ^+++^*p* < 0.001 for NaCl intake in DBA/2NTac, DBA/2NCrl and DBA/2NHsd mice on each indicated day, compared to test day 7.

There were no significant effects of sex detected in the initial repeated measures ANOVA for NaCl consumption, with data grouped on sex, *Taar1* genotype, MA dose and test day. Data for the sexes were combined and the analysis revealed a significant *Taar1* genotype x MA dose × test day interaction [*F*_(4, 172)_ = 30.2, *p* < 0.00001]. For the saline-treated mice (Figure 6A), there was no significant effect of *Taar1* genotype, but there was a significant effect of test day for the amount consumed from the NaCl tube [*F*_(4, 100)_ = 3.9, *p* < 0.01]; however, rather than a reduction in intake, there was a significant increase in intake on the final test day, compared to intake on the initial day (*p* < 0.05). For the 2 mg/kg MA-treated mice (Figure 6B), there was a significant *Taar1* genotype × test day interaction [*F*_(4, 72)_ = 47.9, *p* < 0.00001] that was associated with resistance of homozygous *Taar1*^*m1J*^ mice to MA-induced CTA. Thus, there was no significant change in NaCl consumption across days in these mice, whereas intake significantly decreased across days in *Taar1*^+^ homozygotes (*p* < 0.0001). The *Taar1*^+^ homozygotes consumed significantly less from the NaCl tube, compared to *Taar1*^*m1J*^ homozygotes on all days except test day 7 (the initial test day), at which time NaCl consumption was measured prior to pairing with MA. Correlations were examined between *Taar1* genotype and NaCl intake during the final conditioning day (test day 15) as a measure of CTA. *Taar1* genotype explained 92% of the variance in NaCl consumption in the MA-treated mice. This correlation was not significant for saline-treated mice (Table 3).

### DBA/2 mice

In the initial repeated measures ANOVA for NaCl consumption, with data grouped on sex, vendor, MA dose and test day, there was a significant sex x MA dose interaction [*F*_(1, 47)_ = 4.5, *p* < 0.05], but no other significant main or interaction effects involving sex. The interaction was due to greater overall intake of NaCl in male than female mice, only in the saline-treated animals (*p* < 0.01; mean ± SEM = 2.3 ± 0.1 and 1.9 ± 0.1 ml for males and females, respectively). We next examined the significant 3-way vendor × MA dose × test day interaction [*F*_(12, 220)_ = 2.2, *p* < 0.05]. For the saline-treated mice (Figure 6C), there were no significant effects of vendor or day, indicating similar and stable NaCl intake. For the mice treated with 2 mg/kg MA (Figure 6D), there was a significant vendor x test day interaction [*F*_(12, 112)_ = 5.1, *p* < 0.00001]. There were vendor-specific effects for all days (*p*s < 0.001) except the first day (day 7), when NaCl consumption was measured prior to any MA conditioning. On subsequent days, DBA/2J mice consumed significantly more NaCl than DBA/2 mice from the other three vendors. Furthermore, there were significant reductions in NaCl consumption across days in DBA/2NCrl, DBA/2NTac, and DBA/2NHsd mice, but not DBA/2J mice. When the correlation was examined between *Taar1* genotype and NaCl intake for the final conditioning day (test day 15), *Taar1* genotype explained 69% of the variance in NaCl consumption in the MA-treated mice. This correlation was not significant for saline-treated mice (Table 3).

### *Taar1* genotype and MA effects on core body temperature

The main finding for these studies is that *Taar1* genotype was associated with sensitivity to MA-induced hypothermia, with homozygous *Taar1*^*m1J*^ mice exhibiting resistance.

### BXD RI mice

**Figure 7:**
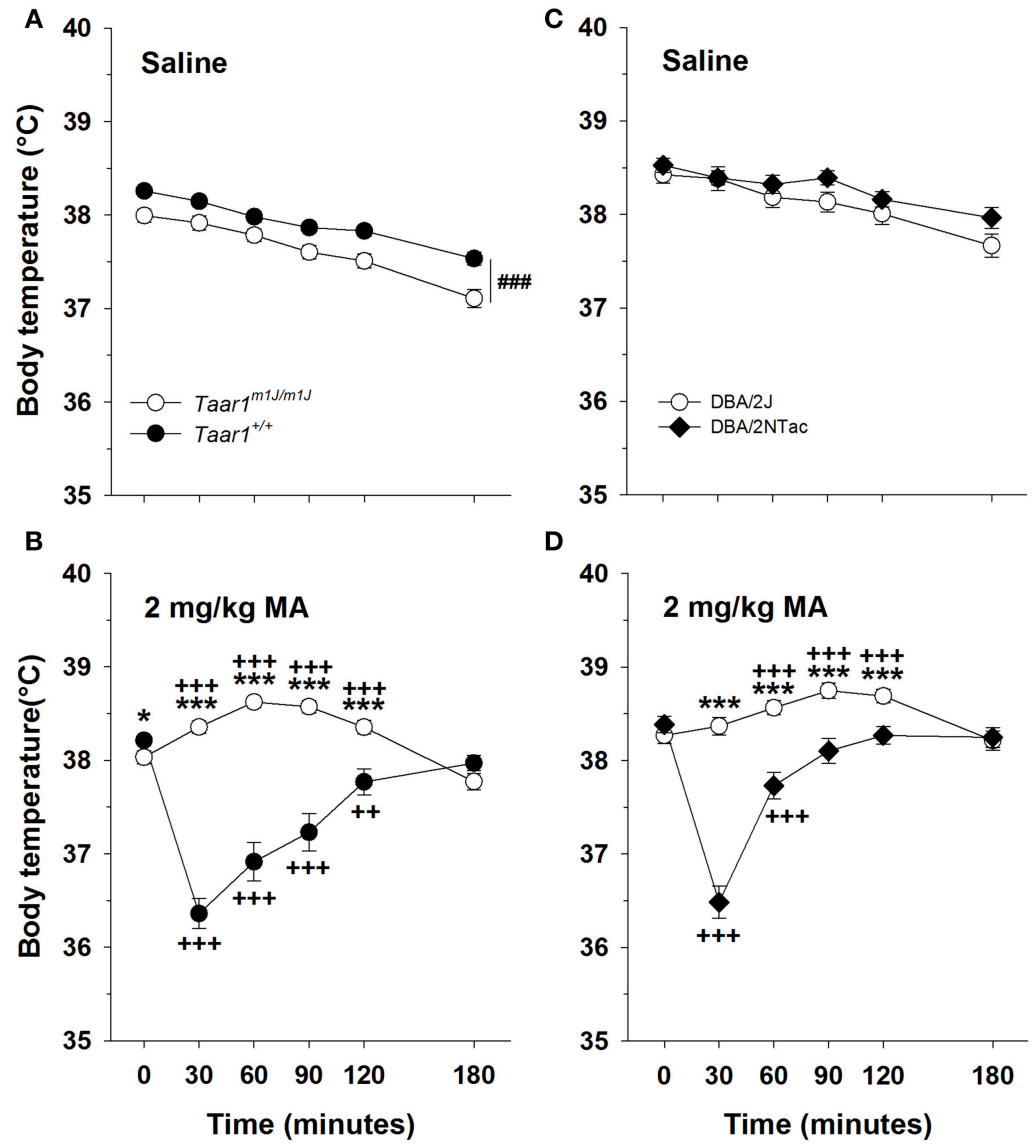
The homozygous *Taar1*^*m1J*^ genotype is associated with resistance to MA-induced hypothermia. Mean ± SEM core temperature immediately before and for 3 h after **(A)** saline or **(B)** 2 mg/kg MA administration in BXD RI mice. Mean ± SEM core temperature immediately before and for 3 h after **(C)** saline or **(D)** 2 mg/kg MA administration in DBA/2J and DBA/2NTac mice. ^###^*p* < 0.001 for the main effect of genotype; ^*^p < 0.05 and ^***^p < 0.001 for the genotype comparison at the indicated time point; ^++^*p* < 0.01 and ^+++^*p* < 0.001 for temperature difference between the indicated time point and T0, within each genotype.

A repeated measures ANOVA on body temperature, with sex, *Taar1* genotype, MA dose and time as factors, did not detect any significant main or interaction effects involving sex, so data were further analyzed for the sexes combined. There was a significant *Taar1* genotype × dose × time interaction [*F*_(5, 1605)_ = 50.7, *p* < 0.0001]. For the saline group (Figure 7A), there were significant main effects of *Taar1* genotype [*F*_(1, 760)_ = 12.6, *p* < 0.001] and time [*F*_(5, 760)_ = 90.2, *p* < 0.0001]. Homozygous *Taar1*^+^ mice had a 0.5°C higher body temperature than homozygous *Taar1*^*m1J*^ mice overall, and temperature declined by 0.8°C over time. For the 2 mg/kg MA group (Figure 7B), there was a significant *Taar1* genotype x time interaction [*F*_(5, 845)_ = 81.0; *p* < 0.0001]. *Taar1*^+^ homozygotes exhibited a hypothermic response to MA and had significantly lower body temperatures at 30–120 min post MA treatment, compared to their body temperature just prior to treatment (T0). *Taar1*^*m1J*^ homozygotes were resistant to the hypothermic effects of MA and exhibited significant hyperthermia after MA treatment at all post-MA administration time points except T180, when their temperature was reduced compared to T0. This reduction was not likely related to MA treatment, as maximal hypothermia occurs at T30 and the mean temperature reduction was only 0.3°C. The disparate responses of the genotypes to MA were supported by significant differences between the *Taar1* homozygote groups at 30–120 min. There was also a smaller, but significant difference at baseline, with the homozygous *Taar1*^+^ group having about a 0.2°C higher body temperature, compared to the homozygous *Taar1*^*m1J*^ group.

Because the most significant change in body temperature was at 30 min post MA treatment here and in previous studies (Harkness et al., 2015), the correspondence between *Taar1* genotype and change in body temperature from baseline at 30 min (T30 minus T0) was examined for the saline and the MA groups (Table 3). For the MA group, the correlation was significant and *Taar1* explained 50% of the variation in thermal response to MA. For the saline group, the correlation was not significant.

### DBA/2J and DBA/2NTac

To examine the relationship between *Taar1* and hypothermic response to MA on a more isogenic background, the DBA/2J and DBA/2NTac strains were compared. Consistent with all other findings, the DBA/2J strain, carrying the mutant *Taar1*^*m1J*^ allele, was resistant to MA-induced hypothermia. A repeated measures ANOVA on body temperature, with sex, vendor, MA dose and time as factors, detected a significant sex × MA dose × time interaction [*F*_(5, 435)_ = 3.6, *p* < 0.01], but no effects of sex involving vendor. Further examination within each dose group revealed a significant time × sex interaction only in the saline group [*F*_(5, 215)_ = 5.3, *p* < 0.0001]. Males had a lower average body temperature than females of about 0.4°C, only at T180. Because this effect was not associated with vendor or MA treatment, a repeated measures ANOVA was performed on data for the sexes combined. There was a significant vendor × MA dose × time interaction [*F*_(5, 455)_ = 20.6, *p* < 0.0001]. For the saline group (Figure 7C), the only significant result was an effect of time [*F*_(5, 225)_ = 25.4, *p* < 0.0001]. For the 2 mg/kg MA group (Figure 7D), there was a significant vendor × time interaction [*F*_(5, 230)_ = 41.6, *p* < 0.0001]. Body temperature was dependent on time in both the DBA/2NTac and DBA/2J mice (*p*s < 0.0001). DBA/2NTac mice displayed significant hypothermia at T30 and T60 post MA treatment, whereas the DBA/2J mice displayed a significant hyperthermic response at T60-120. Compared to DBA/2J mice, DBA/2NTac mice had significantly lower temperatures at all time points except T0 and T180.

The correlation between *Taar1* genotype and change in body temperature from baseline at 30 min (T30-T0) was examined for the saline and the MA groups (Table 3). For the MA group, the correlation was significant and *Taar1* explained 67% of the variation in thermal response to MA. For the saline group, the correlation was not significant.

### Chronology for the *Taar1* mutation

**Figure 8:**
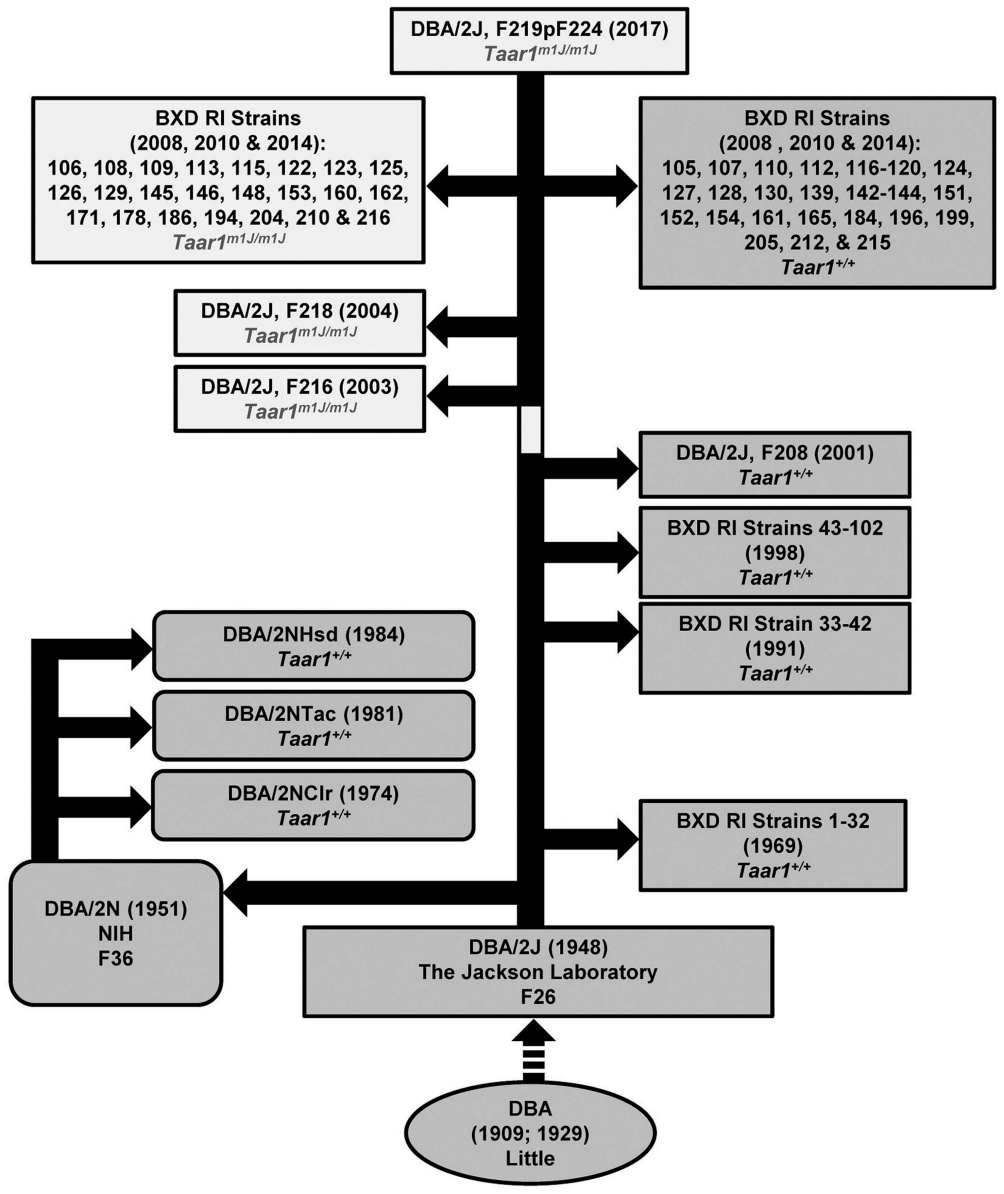
Chronology for the *Taar1*^*m1J*^ allele. The historical sequence for DBA/2 mice is indicated, to the best of our knowledge. Dr. Clarence Cook Little began inbreeding the first DBA strain in 1909. In 1929, The Jackson Laboratory was established by Dr. Little, and strain crosses were initiated from which DBA sublines emerged, including the DBA/2. By 1948, the F26 generation had been produced at The Jackson Laboratory. F36 DBA/2 mice were distributed to the National Institutes of Health (NIH) in 1951, and named DBA/2N. Mice from NIH were distributed to Charles River, Taconic, and Harlan Sprague Dawley (now Envigo) in 1974, 1981, and 1984, respectively, where the mice were renamed to indicate source (DBA/2NClr, DBA/2NTac, and DBA/2NHsd, respectively). Current genotyping of mice sourced from those suppliers indicates that all are homozygous *Taar1*^+^. Multiple waves of BXD RI strains have been initiated beginning ca 1969, ca 1991, ca 1998, ca 2008, ca 2010, and ca 2014 (for details see Taylor et al., 1973, 1975, 1999; Peirce et al., 2004; http://www.genenetwork.org/mouseCross.html). DNA samples were available from many strains and *Taar1* genotyping results are indicated. Some sequencing results for BXD RI strains not used in the current manuscript have been published (Shi et al., 2016). Only the more recently derived BXD RI strains possess the mutant *Taar1*^*m1J*^ allele. Genotyping of DNA from generation F208, F216, and F218 DBA/2J mice indicates that the SNP in *Taar1* at position 229 arose in 2001-2003 (indicated by the light coloration on the main bar of the figure). The dashed arrow indicates that some intermediary events have been omitted. Sequencing results for some additional strains can be found in Shi et al. (2016). Note that among strains BXD1-102, the following were directly sequenced for *Taar1* position 229 genotype: 1, 2, 5, 6, 8, 9, 11, 12, 14–16, 18–25, 27–34, 36, 38–40, 42–45, 48, 48a, 51, 55, 56, 60, 63–66, 68–71, 73–75, 77, 79, 83, 84, 87, 89, 90, and 98–102.

Archived DNA was assayed for the *Taar1* SNP in tissue samples obtained from The Jackson Laboratory for several DBA/2J mice from generations produced between 2001 and 2007. The *Taar1* SNP that is associated with a non-functional receptor was not present in samples from animals prior to May of 2001, but was present in archived samples from DBA/2J mice beginning in 2003. Further investigation by personnel at The Jackson Laboratory provided evidence that mice from breeder pair 03-06347 (used for breeding in 2003) were homozygous for the mutation, which indicates that the *Taar1* SNP arose after May of 2001, and by the time that new breeding stock for the DBA/2J mice was established at The Jackson Laboratory in 2003. The chronology is illustrated in Figure 8.

## Discussion

Results from the current studies strongly implicate an impact of TAAR1 function on MA intake and sensitivity to an aversive and a physiological response to MA. The triad of traits examined here has consistently been identified as genetically correlated in selectively bred MADR mice (Phillips and Shabani, 2015; Phillips et al., 2016), and previous data implicated *Taar1* in the genetic associations (Harkness et al., 2015). The current data confirm the correspondence of higher MA intake, lack of sensitivity to MA-induced CTA and lack of sensitivity to MA-induced hypothermia with a *Taar1* allele that codes for a non-functional receptor. Furthermore, *Taar1* genotype frequency data support rapid fixation of the *Taar1*^*m1J*^ allele in multiple replicates of the MAHDR line and retention of heterozygosity, indicating dominance of the *Taar1*^+^ allele for low MA intake in multiple MALDR lines, and that the *Taar1*^*m1J*^ is not a dominant negative mutation.

The correlation between *Taar1* genotype and each of the MA traits examined here was highly significant on a mixed C57BL/6J × DBA/2J background and an inbred DBA/2 strain background. For the MA intake selection phenotype, the amount of variance for which *Taar1* genotype accounted tended to be greater on the isogenic background. Furthermore, the range of MA intake values for homozygous *Taar1*^*m1J*^ mice tended to be wider for the populations with heterogeneous backgrounds. Thus, the lowest MA intake amount for an inbred DBA/2J mouse was 2.68 mg/kg and there was no overlap in MA intake values between individuals sourced from The Jackson Laboratory and the DBA/2 mice from other vendors that do not possess the *Taar1*^*m1J*^ allele (Figure 5C). However, in the BXD RI mice, several homozygous *Taar1*^*m1J*^ individuals consumed no MA and the intake values for the homozygous *Taar1*^*m1J*^ and *Taar1*^+^ populations overlapped (Figures 4A,B). Overall, the data suggest that polymorphisms associated with the C57BL/6J strain impact MA consumption, either independently or epistatically. These individual differences are of interest, because they suggest that there are individuals with genotypes that can counteract the high risk associated with the *Taar1* mutation, which could be studied to identify novel mechanisms for treatment.

The *Taar1* SNP at position 229 in the DBA/2J strain was traced to a spontaneous event occurring at The Jackson Laboratory in 2001–2003. DBA/2 mice sourced from other common vendors do not possess the SNP and have MA traits characteristic of mice that possess the alternative allele. The current findings predict that for data collected prior to 2001, the C57BL/6 and DBA/2 mice, regardless of vendor, would not have differed for the traits examined here (or perhaps traits measured similarly). There are no published MA drinking data in C57BL/6 or DBA/2 mice prior to ours in 2014; in that study DBA/2J mice consumed more MA than C57BL/6J mice (Eastwood and Phillips, 2014). However, Meliska et al. (1995) published data in C57BL/6NHsd and DBA/2NHsd mice for amphetamine consumption and found significantly greater intake in C57BL/6NHsd females, compared to DBA/2NHsd, the opposite strain difference to ours. This difference was not found in males. Although we predicted that mice of these strains would not differ in meth(amphetamine) intake prior to the appearance of the *Taar1* SNP, the reversal of the strain difference could reflect other alleles in the C57BL/6 strain background that impact this trait in the absence of the *Taar1* polymorphism. In a recent study by Fultz et al. (2017), C57BL/6J mice were offered four bottles of MA of differing concentrations during 2 h of the dark cycle, with no water choice. Total consumption ranged from about 0.3–0.7 mg/kg. Low MA intake levels (up to ~0.18 mg/kg/1 h session) were also found in C57BL/6J mice in an operant oral MA self-administration study (Szumlinski et al., 2017). DBA/2J mice were not included in these studies; however, Sharpe et al. (2014) measured intravenous self-administration of MA in DBA/2J mice during 1-h operant sessions and obtained levels of about 4 mg/kg at the highest infusion dose (0.15 mg/kg/infusion). Also relevant are results for TAAR1 partial agonists. The partial agonist, RO5203648 reduced MA self-administration in rats, while not affecting sucrose self-administration (Cotter et al., 2015). Another partial TAAR1 agonist, RO5263397 reduced the breakpoint for MA self-administration and reduced MA-primed reinstatement of responding after extinction. However, when substituted for MA, the partial agonist did not sustain responding (Pei et al., 2017). These recently published studies support a role for TAAR1 in MA intake and reflect lower voluntary intake in C57BL/6 mice that possess a functional TAAR1, compared to DBA/2 mice that lack TAAR1 function, consistent with our findings.

With regard to meth(amphetamine)-induced body temperature change and CTA, there have been several relevant strain comparison studies, all performed prior to when the *Taar1* SNP arose or in strains from vendors other than The Jackson Laboratory. The earliest of these studies measured temperature after 2, 10, and 20 mg/kg d-amphetamine in DBA/2J and C57BL/6J mice. Both strains exhibited a hypothermic response to the 2 mg/kg dose, consistent with the expectation for animals with functional TAAR1. DBA/2J mice exhibited hyperthermic responses to the higher amphetamine doses that were largely absent in C57BL/6J mice (Seale et al., 1985). Another study examined temperature responses to 4, 8, and 16 mg/kg MA in DBA/2J and C57BL/6J mice, 48 min after treatment. The strains did not exhibit significant thermal responses to any of the MA doses, although there was significant hypothermia or hyperthermia that varied among the BXD RI strains that were also tested (Grisel et al., 1997). Kita et al. (1998) examined thermal responses in DBA/2N and C57BL/6N mice, 1 h after each of four treatments with 2 or 4 mg/kg MA given every 2 h. Hyperthermia was observed in both strains, beginning with the second injection. Responses appeared to be largely comparable, although statistics were not given to allow this to be fully assessed. Thus, results vary considerably across these studies, as did drugs, doses and number of treatments, but agree in general with the prediction that the DBA/2 and C57BL/6 mice did not differ in hypothermic response to amphetamines prior to when the *Taar1* SNP arose. Finally, C57BL/6JICo and DBA/2JICo mice (Charles River, Italy) exhibited comparable 1 and 2 mg/kg MA-induced CTA (Orsini et al., 2004).

Table 4 summarizes the results for the effects of sex in the current studies. For most studies, sex did not have a significant impact. However, for MA intake, a sex difference was found in two of four studies. For the mice chosen as breeders of the MADR lines, females of the higher MA-intake homozygous *Taar1*^*m1J*^ genotype consumed about 15% more MA than males. A similar result was obtained in the BXD RI mice, but not in the F2 or DBA/2 inbred mice. We have observed a significant sex difference in MA intake in some (Wheeler et al., 2009; Shabani et al., 2011, 2016), but not all (Eastwood and Phillips, 2014; Harkness et al., 2015; Shabani et al., 2016) of our prior studies. When a difference has been found, females have consumed more MA than males and the difference has been of about the same magnitude as reported here. A recent study in humans, including 413 males and 369 females who use MA, reported that females had heavier, more frequent and greater lifetime episodes of MA use and were more likely to be MA-dependent and to experience withdrawal than males (Rungnirundorn et al., 2017). Although there is some evidence for more MA intake in females in the rodent studies, the sex effect has been modest. For other traits, sex effects were inconsistently found.

**Table 4:** Sex-dependent effects.

	**F2 mice**	**MADR mice**	**BXD RI mice**	**DBA/2 mice**
MA intake (mg/kg)	F = M	F > M (~15%)	F > M (~15%)	F = M
Total volume (ml)	F = M	F = M	F = M	F = M
CTA: NaCl consumption 2 mg/kg MA			F = M	F = M
CTA: NaCl consumption saline group			F = M	F < M
Thermal response 2 mg/kg MA			F = M	F = M
Thermal response saline group			F = M	F > M

Total volume consumed was examined in the MA intake studies to determine whether fluid intake was associated with level of MA intake. In addition, the association of *Taar1* genotype with total volume was examined. In the F2 and MADR mice, *Taar1* genotype was positively associated with total volume consumed; thus the homozygous *Taar1*^*m1J*^ genotype was associated with greater total volume. *Taar1* genotype explained 5–19% of the variance in total volume consumed during the time when MA was offered. However, there was no impact of *Taar1* genotype on water intake during the 2 days prior to MA access. Since greater total volume was associated with the higher MA intake, homozygous *Taar1*^*m1J*^ genotype, it is possible that stimulant effects of MA increase overall activity and generate thirst. However, this effect was not found in BXD RI or DBA/2 mice from the various vendors, and MAHDR mice that consumed an average of about 13 mg/kg MA in a binge drinking model did not consume more total volume than MALDR mice that consumed an average of 1 mg/kg MA (Shabani et al., 2016). In our selection studies, we have sometimes found the MAHDR line to consume about 0.5 ml more total volume than the MALDR line (Wheeler et al., 2009; Shabani et al., 2011). In general, *Taar1* associations with total volume are not consistent and are modest compared to associations with MA intake.

A high resolution gene map was generated in 2006 for the BXD RI strains (Shifman et al., 2006) and 52 SNPs were identified as new mutations for which variation corresponded with the phases during which the strains were derived. There were three phases of BXD RI development prior to 2006 (derived beginning in 1969, 1991, and 1998); the *Taar1* SNP was not among the 52 identified SNPs, since it arose in 2001–2003. However, the former analysis confirms that spontaneous mutations are not rare. For example, a spontaneous mutation in *Gpnmb*, a gene that contributes to pigmentary-related eye diseases, is known to have been fixed in the DBA/2J stock (Libby et al., 2005; Lu et al., 2011) and an in-frame deletion in centrosomal/ciliary protein *Cep290* that produces retinal degeneration was fixed in BXD24 (Chang et al., 2006). In the case of the *Taar1* SNP, we have determined that the mutation is a non-synonymous substitution that alters the amino acid sequence and function of the resulting receptor, as well as having a significant impact on several MA-related traits. *Taar1* is located on mouse chromosome 10 in a cluster of *Taar* genes (from 23,920,387 to 24,109,564 base pairs) and the SNP in *Taar1* at position 229 is the only sequence variant between the reference C57BL/6J strain and the DBA/2J strain for this entire cluster of genes (Shi et al., 2016).

Unlike the receptor expressed by *Taar1*^+^, the receptor expressed by *Taar1*^*m1J*^ does not respond *in situ* to MA, or to the trace amines, β-phenethylamine (β-PEA) or tyramine (Harkness et al., 2015; Shi et al., 2016). Thus, the spontaneously occurring SNP created a functional TAAR1 knockout for which a receptor is expressed, but not stimulated by agonists to elicit a cAMP response. We and others have generated data in classical knockout mice in which the *Taar1* gene was genetically altered via homologous recombination and does not produce a receptor protein. Results in knockout mice for MA-related traits have been similar to those for the MAHDR mice (Achat-Mendes et al., 2012; Harkness et al., 2015), suggesting that the trait alterations are not dependent upon the expression of the non-functional protein. However, it remains possible that the receptor expressed by the *Taar1*^*m1J*^ allele has some function other than that regulated by agonist binding. Also remaining to be studied are the potential functional and behavioral consequences of the full slate of almost 50 non-synonymous SNPs reported for the human-*TAAR1* (dbSNP database, NCBI). Shi et al. (2016) examined agonist-stimulated cAMP production for eight human receptor variants by constructing them into the *TAAR1* reference human sequence and transfecting them into CHO-K1 cells. All variants expressed a receptor, and a cAMP response to β-PEA was observed in cells transfected with the reference sequence, whereas a reduced cAMP response or no response was observed for other variants. There is the possibility that these genetic variants could impact response to MA, risk for a MA use disorder, or response to treatment. In theory, individuals carrying non-functional variants should be at greatest risk, but would not be subject to treatment with TAAR1 agonists or partial agonists. However, it is possible that those with greatly reduced TAAR1 function could benefit from TAAR1-targeted treatments (Sotnikova et al., 2009; Cotter et al., 2015; Jing and Li, 2015; Edelmann et al., 2016; Pei et al., 2016; Phillips et al., 2016).

## Author contributions

CR: Development of experimental protocols, supervision of technical support, statistical analysis, interpretation of data, and wrote the manuscript interactively with TP. HB, ZZ, JE, JM, and NV: Data acquisition and entry, and checking of entered data for accuracy. RW: Provided some mouse breeding stock and assisted in data interpretation and manuscript editing. TP: Experimental design, analysis and interpretation of all data, and wrote the manuscript interactively with CR.

### Conflict of interest statement

The authors declare that the research was conducted in the absence of any commercial or financial relationships that could be construed as a potential conflict of interest.
